# Machine‐learning‐based prediction of respiratory flow and lung volume from real‐time cardiac MRI using MR‐compatible spirometry

**DOI:** 10.1002/mp.18019

**Published:** 2025-08-11

**Authors:** Halima Malik, Tobias Uelwer, Lena Maria Röwer, Janina Hußmann, Pablo Emilio Verde, Stefan Harmeling, Dirk Voit, Jens Frahm, Dirk Klee, Frank Pillekamp

**Affiliations:** ^1^ Department of Diagnostic and Interventional Radiology Medical Faculty and University Hospital Düsseldorf, Heinrich Heine University Düsseldorf North Rhine‐Westphalia Germany; ^2^ Department of Computer Science Technical University of Dortmund Dortmund North Rhine‐Westphalia Germany; ^3^ Coordination Centre for Clinical Trials Heinrich Heine University Düsseldorf North Rhine‐Westphalia Germany; ^4^ Biomedical NMR, Max Planck Institute for Multidisciplinary Sciences Göttingen Lower Saxony Germany

**Keywords:** cardiac MRI, machine learning, real‐time MRI, respiration

## Abstract

**Background:**

Cardiac real‐time MRI (RT‐MRI) in combination with MR‐compatible spirometry (MRcS) offers unique opportunities to study heart‐lung interactions. In contrast to other techniques that monitor respiration during MRI, MRcS provides quantitative respiratory data. Though MRcS is well tolerated, shortening of the scanning time with MRcS would be desirable, especially in young and sick patients.

**Purpose:**

The aim of the study was to predict airflow and lung volume based on RT‐MR images after a short learning phase of combined RT‐MRI and MRcS to provide respiratory data for subsequent short axis stack‐based volumetries.

**Methods:**

Cardiac RT‐MRI (1.5 T; short axis; 30 frames/s) was acquired during free breathing in combination with MRcS in adult healthy subjects (*n* = 10). MR images with MRcS were recorded during a learning phase to collect training data. The iterative Lucas‐Kanade method was applied to estimate optical flow from the captured MR images. A ridge regression model was fitted to predict airflow and thus also the lung volume from the estimated optical flow. Hyperparameters were estimated using leave‐one‐out cross validation and the performance was assessed on a held‐out test dataset. Different durations and compositions of the learning phase were investigated to develop the most efficient measurement protocol. Coefficient of determination (R^2^), relative mean squared error (rMSE), Bland‐Altman analysis on absolute tidal volume difference (aTVD), and absolute maximal airflow difference (aMFD) were used to validate the predictions on held‐out test data.

**Results:**

MRI combined with MRcS can train a machine learning algorithm to provide excellent predictive quantitative respiratory volume and flow for the remaining study. The optimal trade‐off between predictive power and time necessary for training was reached with a shortened cardiac volumetry protocol covering only about two breaths per slice and every second slice (airflow: mean R^2^: 0.984, mean rMSE: 0.015, Bias aMFD: ‐0.01 L/s with +0.084/‐0.1 95% CI and volume: mean R^2^: 0.990, mean rMSE: 0.003, Bias aTVD: 4.27 mL with +33/‐24 95% CI) at a total duration of 100 s. Shorter protocols or application of the algorithm to subsequent studies in the same subject or even in different subjects still provided useful qualitative data.

**Conclusion:**

Machine‐learning‐based prediction of respiratory flow and lung volume from cardiac RT‐MR images after a short training phase with MRcS is feasible and can help to shorten the time with MRcS while providing accurate respiratory data during RT‐MRI.

## 1 BACKGROUND

Real‐time MRI (RT‐MRI) is a fast‐imaging technique that uses highly undersampled radial data encoding in combination with nonlinear inverse reconstruction. Its high frame rate (20–50 frames per second) allows high‐quality imaging during free breathing.^1^ In sick patients or small children who are not able to hold their breath, free breathing is of major importance to avoid intubation narcosis. However, breathing modifies cardiac position, function, and dimensions significantly. The most pronounced respiratory effect on the latter is an increase in right ventricular end‐diastolic volume and right ventricular stroke volume.^2^

Accordingly, to conduct quantitative cardiac image analyses (e.g., volumetry), it is essential to have a respiratory signal. For quantitative studies on heart‐lung interactions, even a method to measure lung volume and airflow quantitatively is mandatory. Navigator echoes cannot be acquired during RT‐MRI for technical reasons, while the frequently used detection of the respiratory expansion of thorax or abdomen by a belt, bellows or cushion is not quantitative.

Spirometry is the gold standard to measure tidal volumes and airflow and is regularly used in adult and pediatric pulmonology. It has been shown to be feasible during MRI.^3^ Though magnetic resonance‐compatible spirometry (MRcS) is quite well tolerated,^4^ for longer time periods it remains somewhat uncomfortable. Therefore, shortening the scanning time with MRcS would be desirable, especially for young children or patients with dyspnea.

For these reasons, the aim of this proof‐of‐principle study was to test to which extent the duration of MRcS could be reduced. We hypothesized that using machine learning and a short learning phase with combined MRcS and MRI, quantitative respiratory data could be generated for the remaining study from respiratory changes detected in MR images and tested whether such an algorithm could even be useful in successive studies of the same or another patient.

## 2 METHOD

Data from 10 adult (six female and four male) volunteers were acquired. None had prior health restrictions. Each volunteer was educated towards MRI functionality, filled out an information sheet and signed a written consent to the outline of our study. The study was approved by the ethics committee of the University Hospital Düsseldorf, study number 6176R. The study was conducted in accordance with the Helsinki Declaration as revised in 2013.

### 2.1 Image and acquisition of physiological data

MR images were recorded using a 1.5 T scanner (MAGNETOM Avanto fit, Siemens Healthcare, software version syngo MR VE11) which included a 32‐channel spine matrix coil and an 18‐channel thorax coil. Each volunteer was placed in a feet‐first supine position.

The cardiac imaging protocol was identical to the protocol used in previously published experiments.^4^ Standard cardiac localizers and retrospectively gated two‐chamber and four‐chamber views were used to define a standardized short‐axis stack for each subject. In accordance with the MRI protocol parameters detailed in Table 1, two datasets were obtained per subject: one comprising a single midventricular slice and the other a complete short‐axis stack for cardiac volumetry. Simultaneously, ECG and respiratory bellows signals were recorded using the Siemens Physiologging (VE11C).

**TABLE 1:** MR‐Parameters. Sequence parameters for real‐time magnetic resonance imaging (MRI) used for method development (midventricular slice, short axis stack volumetry) and resulting parameters for optimized training.

**Sequence parameters**	**Volumetry**	**Midventricular slice**	**Optimized training**
Sequence type	b‐SSFP		
TR/TE (ms)	3.7/1.85		
Orientation	short axis		
Flip angle (°)	60		
Bandwidth (Hz/pixel)	760		
FOV (mm)	320–400		
Image matrix (pixels)	200 × 200		
In‐plane resolution (mm × mm)	1.6 × 1.6		
Slice thickness (mm)	8		
Image acquisition time (ms)	33		
Number of slices	19	1	10
Interslice gap (mm)	0	0	8
Phases	900	4000	300
Total scan duration (s)	570	133	100
Breathing type	free breathing	deep & free breathing	free breathing

Abbreviations: b‐SSFP, balanced steady‐state free precession; FOV, field of view; TE, echo time; TR, repetition time.

### 2.2 Spirometry

**FIGURE 1:**
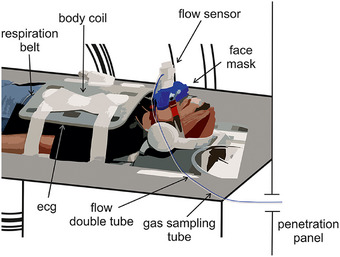
Physiological monitoring. The flow sensor measures the airflow based on the differential pressure on a membrane. Differential pressure information is transported by the flow double tube (blue). A gas sampling tube (white) provides gas for O_2_/CO_2_ measurements. Both tubes are connected to the spirometry unit located in the adjacent control room.

A silicone face mask (COSMED Deutschland GmbH, Werneck, Germany, size S) was connected to the non‐magnetic light‐weight flow sensor. This sensor measures the airflow based on the differential pressure on a membrane. The differential pressure information is transported via a modified 6 m double tube to the spirometry unit (Geratherm Respiratory GmbH, Bad Kissingen, Germany) outside the scanner (Figure 1). Its effective dead space is less than 96 mL, which has been demonstrated to be tolerable. Furthermore, the tube set included a pipe for humidity balance (Perma Pure, Lakewood, NJ, USA), a hydrophobic mini filter and an adapter for the connection to the flow sensor. Airflow data were recorded with a sampling rate of 125 Hz with an accuracy of ± 3% or ± 50 mL/s and a range from ± 5 L/s for ventilation.

Removing and even repositioning the face mask can be performed easily inside the MR‐Scanner and without changing the position of the patient table (Video S1).

### 2.3 Respiratory model

#### 2.3.1 Training and test datasets

**FIGURE 2:**
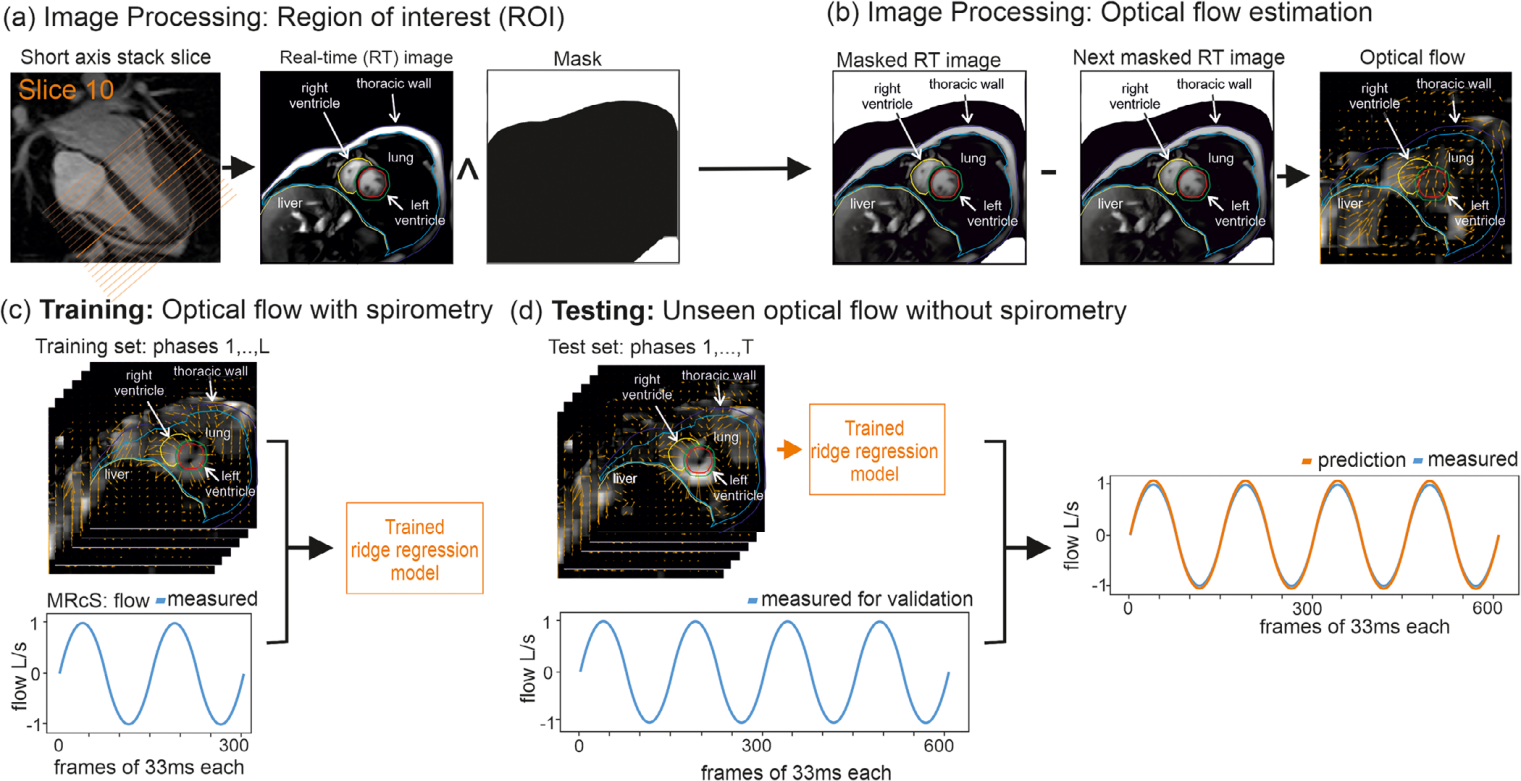
Respiratory model. To limit the analysis to breathing‐affected pixels a mask was overlayed (a). To estimate optical flow 10 steps of the iterative Lucas‐Kanade method were applied to consecutive subsampled masked images (b). Estimated optical flow and spirometry‐measured airflow were used to fit a linear model using ridge regression (c). Estimated optical flow of unseen images, which were provided to the fitted model, were used to predict airflow (d).

The recorded respiratory flow measurements and the corresponding MR images were split into a training dataset, which consisted of L samples, and a test dataset, which consisted of T samples. Overall, at most one third of the total data acquired was used for model training (Figure 2c). The remaining test data was used to evaluate the model (Figure 2d).

#### 2.3.2 Preprocessing

The recorded respiratory flow measurements $f^{\left(1\right)},\text{…},f^{\left(L\right)}\in R$ were normalized by

$$f_{\text{norm}}^{\left(l\right)}=\frac{f^{\left(l\right)}-\mu_{f}}{\sigma_{f}}\;\mathrm{for}\;l=1,\cdots ,L,$$

where $\mu_{f}$ and $\sigma_{f}$ were the mean and the standard deviation of $f^{\left(1\right)},\text{…},f^{\left(L\right)}$.

#### 2.3.3 Model architecture

Each MR image consisted of 200×200 pixels. A mask was applied to limit the region of interest to pixel effected by breathing (Figure 2a). In a second step, each masked image was downsampled to a resolution of 40 × 40 pixels. The downsampled masked MR images were used to calculate the normalized optical flow, represented as vectors with two entries, by applying 10 steps of the iterative Lucas‐Kanade method.^5^, ^6^ The implementation provided by scikit‐image^7^ was used. The optical flow represented a sequence of tensors $X^{\left(1\right)},\text{…},X^{\left(L\right)}\in R^{40\times 40\times 2},$ where the flow at each pixel of the subsampled masked MR image was described by two values (Figure 2b). Normalized airflow measurements were predicted using a linear model

$${\hat{f}}_{\text{norm}}^{\left(l\right)}=\sum_{m=1}^{40}\sum_{n=1}^{40}\sum_{k=1}^{2}W_{\mathrm{mnk}}X_{\mathrm{mnk}}^{\left(l\right)}+b,$$

where $W\in R^{40\times 40\times 2}$ and b∈R were learnable model parameters.

#### 2.3.4 Model fitting

Parameters W and b were estimated using ridge regression, which solved the regularized least‐squares problem

$$\min_{W,b}\sum_{l=1}^{L}{\left({\hat{f}}_{\text{norm}}^{\left(l\right)}-f_{\text{norm}}^{\left(l\right)}\right)}^{2}+\lambda \sum_{m=1}^{40}\sum_{n=1}^{40}\sum_{k=1}^{2}W_{\mathrm{mnk}}^{2},$$

where λ>0 was a hyperparameter that was chosen using leave‐one‐out cross‐validation, which was applied to the training set. Scikit‐learn^8^ was used to fit the model and perform the cross‐validation in Python (Figure 2c).

#### 2.3.5 Prediction and postprocessing

MR images from the test dataset were used to estimate optical flow tensors $X^{\left(1\right)},\text{…},X^{\left(T\right)}$, which were subsequently fed into our fitted model to calculate airflow predictions ${\hat{f}}_{\text{norm}}^{\left(1\right)},\cdots ,{\hat{f}}_{\text{norm}}^{\left(T\right)}$ (Figure 2d).

Next, the airflow predictions ${\hat{f}}_{\text{norm}}^{\left(1\right)},\cdots ,{\hat{f}}_{\text{norm}}^{\left(T\right)}$ were denormalized

$${\hat{f}}_{\text{denorm}}^{\left(t\right)}=\sigma_{f}\;{\hat{f}}_{\text{norm}}^{\left(t\right)}+\mu_{f\;}\;\mathrm{for}\;t=1,\cdots ,T.$$

Additionally, ${\hat{f}}_{\text{denorm}}^{\left(1\right)},\cdots ,{\hat{f}}_{\text{denorm}}^{\left(T\right)}$ were smoothed using the Savitzky‐Golay Filter^9^ implemented by SciPy.^10^ In the following the denormalized smoothed airflow predictions are denoted as ${\hat{f}}^{\left(1\right)},\text{…},{\hat{f}}^{\left(T\right)}$ and were used as the predicted airflow estimation in further analysis.

Volume measurements $v^{\left(1\right)},\text{…},v^{\left(T\right)}$ and volume predictions ${\hat{v}}^{\left(1\right)},\text{…},{\hat{v}}^{\left(T\right)}$ were calculated using airflow measurements $f^{\left(1\right)},\text{…},f^{\left(T\right)}$ and denormalized filtered airflow predictions ${\hat{f}}^{\left(1\right)},\text{…},{\hat{f}}^{\left(T\right)}$ as

$$v^{\left(t\right)}=c\sum_{k=1}^{t}f^{\left(k\right)}\;\mathrm{for}\;t=1,\cdots ,T,$$

$${\hat{v}}^{\left(t\right)}=c\sum_{k=1}^{t}{\hat{f}}^{\left(k\right)}\;\mathrm{for}\;t=1,\cdots ,T,$$

where c = 10 is a constant used to scale data to the correct magnitude of volume measurements and predictions in mL. Subsequently a baseline correction was performed.

### 2.4 Model configuration

#### 2.4.1 Initial model

**FIGURE 3:**
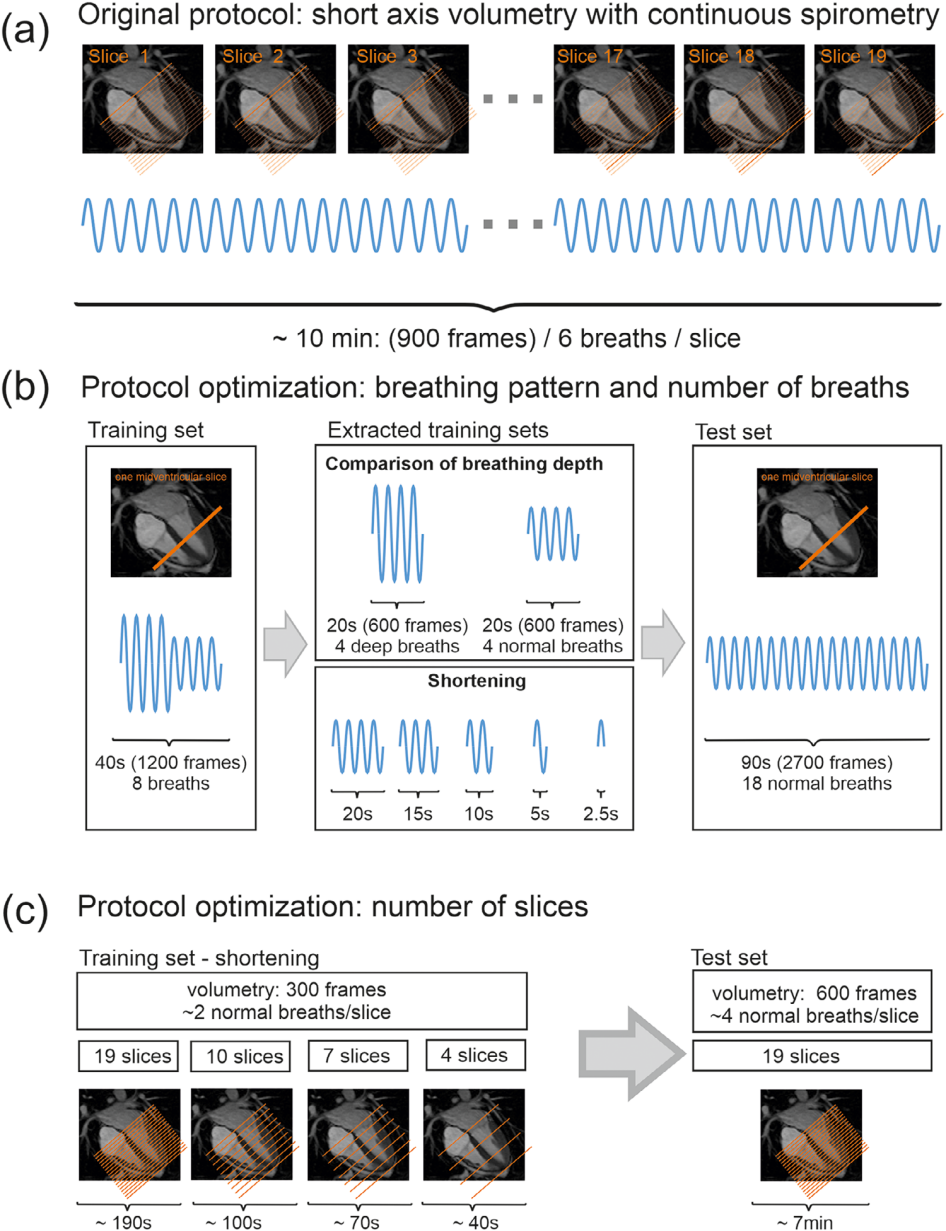
Method development. To predict respiratory measurements of a standard cardiac short axis stack volumetry with free breathing (a), a training set was developed regarding (b) breathing pattern and number of breaths and (c) number of slices selected for training.

The initial cardiac volumetry protocol consisted of the simultaneous acquisition of RT‐MRI and respiratory data during free breathing using MRcS, as shown in Table 1 and Figure 3a. The model configuration aimed to minimize the RT imaging duration required for training with MRcS, while retaining sufficient information to predict respiratory data for a complete short‐axis stack cardiac volumetry.

#### 2.4.2 Assessment of breathing pattern and number of breaths for training

To assess the effect of breathing patterns and the number of breaths used for training, a dataset from a single midventricular slice was evaluated (Table 1). The dataset was split into a fixed test set containing 18 normal breathing cycles (2800 frames, 94 s) and a variable training set consisting of up to four deep and four normal breathing cycles (1200 frames, 40 s) (Figure 3b).

Subsequently, the training set was manually shortened according to breathing pattern and number of breaths, and predictions on the held‐out test data were compared. Specifically, a training set comprising four deep breaths (600 frames, 20 s) was compared to one consisting of four normal breaths (600 frames, 20 s). The effect of reducing the number of breaths was then assessed by analyzing training set configurations containing four, three, two, or one normal breath, as well as a single inspiratory phase (ranging from 600 to 75 frames, or 20 to 2.5 s) (Figure 3b).

#### 2.4.3 Assessment of the number of slices used for training

To evaluate the impact of reduced training time on the prediction of a complete cardiac volumetry, the effect of varying the number of slices used for training on model performance was assessed. The initial dataset consisted of 19 slices (900 frames per slice, 570 s total), which was divided into a fixed test set (600 frames per slice, 380 s total) and variable training configurations (300 frames per slice, up to 190 s total).

Four training protocols were compared based on the number of slices used for training. In accordance with the technical capabilities of the MR scanner, training was conducted using every slice (19 slices, 190 s total, 0 mm interslice gap), every second slice (10 slices, 100 s total, 8 mm interslice gap), every third slice (7 slices, 70 s total, 16 mm interslice gap), and every sixth slice (4 slices, 40 s total, 40 mm interslice gap). For test slices not included in the training set, adjacent slices were used for training, as shown in Figure 3c.

#### 2.4.4 Training set reusability on unknown subject

The reusability of the training set was investigated by analyzing the extent to which a trained model from known subjects could predict respiratory measurements in a test set of one unknown subject. An iterative approach was used to combine known subjects to find common denominators. Therefore, one unknown subject was randomly chosen to provide data for testing, this subject was excluded from the following training data mentioned. As for the training data, singular subjects were selected and a cardiac volumetry of 19 slices (300 frames per slice, 190 s total) was used for training. Regarding the predictive outcome of the trained model on the test set, the training data and test data were assessed, and common denominators such as height, weight, and image orientation were analyzed. Further, complete volumetries of different subjects for training were combined while regarding said common denominators. Lastly, all nine training subjects recorded without regard to common denominators were combined.

In a second setting for one instance in which image orientation of training and test subject did not align, the images of the unknown test set were rotated to fit the image orientation of the training set. For rotation, implementations provided by SciPy^10^ and scikit‐image^7^ in Python were used and added to the respiratory model described in 2.3.2.

Alternatively, a method for adaptive denormalization incorporating the mean and SD of the unknown subject was tested (for details, see Table S4).

#### 2.4.5 Training set reusability on a second scan of the same subject

The potential of reusing a training set to predict respiration during a second scan conducted at a different time for the same subject using an identical MRI protocol was examined. Therefore, for one subject a second test set was recorded, comprising an additional version of the initial cardiac volumetry with 19 slices (900 frames per slice, 570 s total). The training set consisted of the shortened version of the first recorded initial cardiac volumetry as described in Section 2.4.3 with 10 training slices (300 frames per slice, approximately two normal breaths, 100 s total, 8 mm interslice gap). Similarly, as described in 2.4.4 in further analyses images of the second scan were rotated to fit image orientation of the first scan.

### 2.5 Evaluation metrics

Data analysis was conducted in Python.

#### 2.5.1 Qualitative evaluation

For qualitative validation the predicted airflow and lung volume graphs were compared with the actual respiratory measurement graphs collected by MRcS. Further, for each subject the regional contribution in form of weights for each fitted model was analyzed.

#### 2.5.2 Quantitative evaluation

For quantitative evaluation, the relative mean squared error

$${\text{rMSE}}_{\text{flow}}=\frac{\frac{1}{T}\sum_{t=1}^{T}{\left(f^{\left(t\right)}-{\hat{f}}^{\left(t\right)}\right)}^{2}}{\frac{1}{T}\sum_{t=1}^{T}{f^{\left(t\right)}}^{2}}$$

between the flow measurements $f^{\left(1\right)},\text{…},f^{\left(T\right)}$ and their predictions ${\hat{f}}^{\left(1\right)},\text{…},{\hat{f}}^{\left(T\right)}$ was computed. Analogously, the relative mean squared error

$${\text{rMSE}}_{\text{volume}}=\frac{\frac{1}{T}\sum_{t=1}^{T}{\left(v^{\left(t\right)}-{\hat{v}}^{\left(t\right)}\right)}^{2}}{\frac{1}{T}\sum_{t=1}^{T}{v^{\left(t\right)}}^{2}}$$

between the corresponding volume measurements $v^{\left(1\right)},\text{…},v^{\left(T\right)}$ and their predictions ${\hat{v}}^{\left(1\right)},\text{…},{\hat{v}}^{\left(T\right)}$ was calculated.

To further assess the goodness of fit of our model, the coefficient of determination

$$R_{\text{flow}}^{2}=1-\frac{\sum_{t=1}^{T}{\left(f^{\left(t\right)}-{\hat{f}}^{\left(t\right)}\right)}^{2}}{\sum_{t=1}^{T}{\left(f^{\left(t\right)}-\bar{f}\right)}^{2}}$$

for the predicted flow was computed, where $\bar{f}=\frac{1}{T}\;\sum_{t=1}^{T}f^{\left(t\right)}$ denotes the mean of the flow measurements. Analogously, the coefficient of determination

$$R_{\text{volume}}^{2}=1-\frac{\sum_{t=1}^{T}{\left(v^{\left(t\right)}-{\hat{v}}^{\left(t\right)}\right)}^{2}}{\sum_{t=1}^{T}{\left(v^{\left(t\right)}-\bar{v}\right)}^{2}}$$

for the predicted volume was calculated, where $\bar{v}=\frac{1}{T}\;\sum_{t=1}^{T}v^{\left(t\right)}$ denotes the mean of the volume measurements.

For clinical applicability differences in tidal volumes (TV) and maximal airflow (MF) were defined. For every breath a pair of peaks $z^{\left(p\right)}=\left(v^{\left(p\right)},\;{\hat{v}}^{\left(p\right)}\right)$ for p = 1,…,P was chosen and the absolute tidal volume difference

$${\text{aTVD}}_{z^{\left(p\right)}}=\left|{\text{TV}}_{v^{\left(p\right)}}-{\text{TV}}_{{\hat{v}}^{\left(p\right)}}\right|,$$

$$\begin{array}{ccc} & & \mathrm{where}\;{\text{TV}}_{v^{\left(p\right)}}=\left|\max v^{\left(p\right)}-\min\;v^{\left(p\right)}\right|\\ & & \;\text{and}\;{\text{TV}}_{{\hat{v}}^{\left(p\right)}}=\left|\max\;{\hat{v}}^{\left(p\right)}-\min\;{\hat{v}}^{\left(p\right)}\right|, \end{array}$$

between measured volume $v^{\left(1\right)},\text{…},v^{\left(P\right)}$ and predicted volume ${\hat{v}}^{\left(1\right)},\text{…},{\hat{v}}^{\left(P\right)}$ was determined.

For every breath a pair of peaks $z^{\left(p\right)}=\left(f^{\left(p\right)},\;{\hat{f}}^{\left(p\right)}\right)$ for p = 1,…,P was selected and the absolute maximal flow difference

$${\text{aMFD}}_{z^{\left(p\right)}}=\left|\max\;f^{\left(p\right)}-\max\;{\hat{f}}^{\left(p\right)}\right|$$

between measured flow $f^{\left(1\right)},\cdots ,f^{\left(P\right)}$ and predicted flow ${\hat{f}}^{\left(1\right)},\cdots ,{\hat{f}}^{\left(P\right)}$ was evaluated.

For each subject, the mean, the standard deviation (SD), and the minimum‐to‐maximum range of rMSE, R^2^, aTVD and aMFD across all breaths within the test dataset were calculated. Additionally, the overall mean, the SD, and the minimum‐to‐maximum range of the subjects’ means of rMSE, R^2^, aTVD, and aMFD were evaluated. Prediction variability of R^2^ between subjects and between slices was analyzed.

For statistical analysis, agreement between predicted and measured respiratory data was evaluated using Bland‐Altman plots, comparing absolute tidal volume and absolute maximal airflow. For each slice of every subject, one randomly selected pair of predicted and measured absolute TV and predicted and measured absolute MF was chosen, cumulatively illustrated, and evaluated. In each plot the distribution of selected pairs, the 95% confidence interval and the zero line of no bias were analyzed.

The criterion for good agreement in respiratory volume (quality criterion, QC_volume_) was defined as a maximum mean aTVD of less than 20 mL, which is far below the threshold for clinical applications (100 mL for tidal volumes < 1 L).^11^ The quality criterion for airflow (QC_flow_) was defined as a maximum mean aMFD of ± 50 mL/s to ± 100 mL/s.^11^ Predictions exceeding these thresholds for mean aTVD or mean aMFD were considered quantitatively poor. Model configurations were considered unsuitable if quality criteria were not met by at least nine subjects.

## 3 RESULTS

The study was conducted on 10 subjects (six female and four male). The mean age of subjects was 25.4 ± 2.9 years (range: 21 to 30 years), mean body weight 67.6 ± 11.8 kg (range: 54 to 93 kg) and mean height 173.1 ± 8.5 cm (range: 157–185 cm).

**FIGURE 4:**
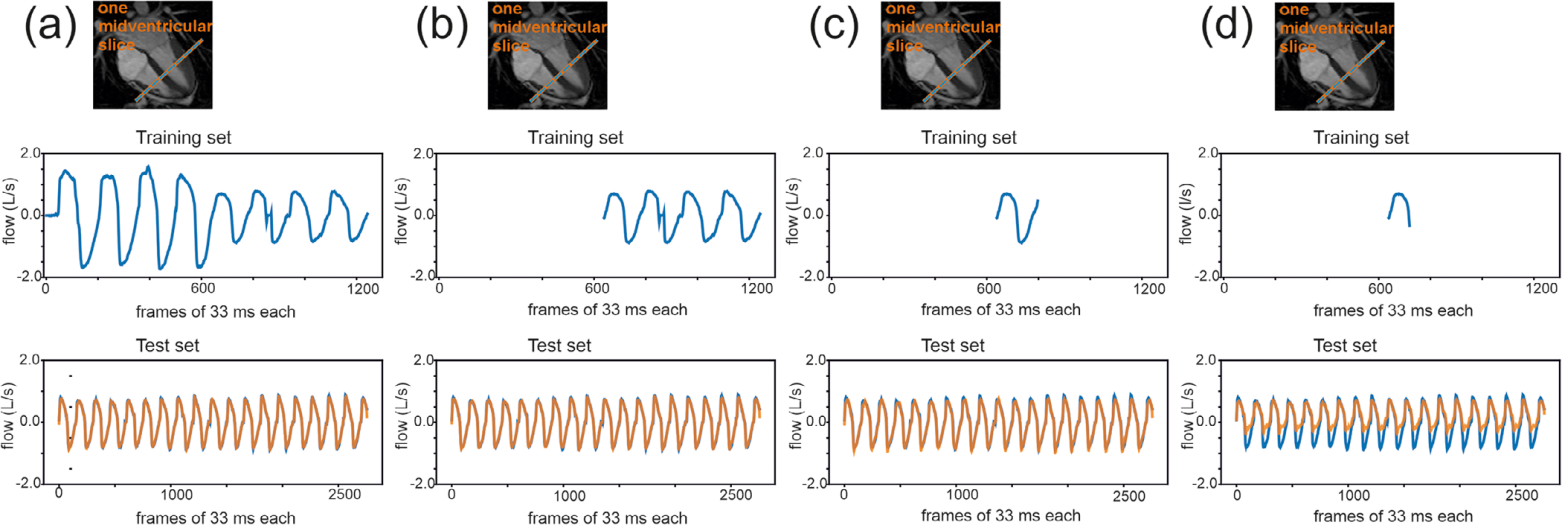
Shortening training. Comparison of the predicted airflow curves (orange) with the measured airflow (blue) for progressively shortened training datasets: (a) four deep and four normal breaths, (b) four normal breaths, (c) one normal breath, and (d) on one inspiratory phase only.

To analyze the predictive properties of our model, the model was trained on one midventricular slice with eight breaths (four deep and four normal breaths, that is, 40 s per slice) (Figure 3b). The prediction was excellent (Figure 4a and Table S1). The predefined quality criteria (QC_volume_ and QC_flow_) were fulfilled in every subject (Table S1). However, the duration of this training phase would be very long (> 760 s). This motivated further analyses for optimization by assessing different breathing patterns and reducing the number of breaths and slices used to train the model.

### 3.1 Optimization of breathing pattern and number of breaths for training

To optimize the breathing pattern and the number of breaths used for training, the predictive outcome of different training sets on one midventricular slice was analyzed. Training sets with four deep and four normal breaths as shown in Figure 4a, four normal breaths as illustrated in Figure 4b and even a single normal breath as depicted in Figure 4c revealed matches of predicted and measured data curves without shift and a good agreement of the curves. In contrast, the reduction of training to a single inspiration did not reach the peaks in predicted volume because of the missing negative values in predicted airflow (Figure 4d).

The quantitative analysis confirmed this observation. A normal breathing pattern and a reduction to two or even a single normal breath for the training set still resulted in accurate mean values (Table S1). Therefore, to cover scan time of at least one normal breath, a scan time covering approximately two normal breaths was necessary to guarantee a sufficient predictive quantitative outcome. As expected, a further reduction did not provide useful quantitative information anymore (Table S1).

### 3.2 Optimization of the number of slices used for training

**FIGURE 5:**
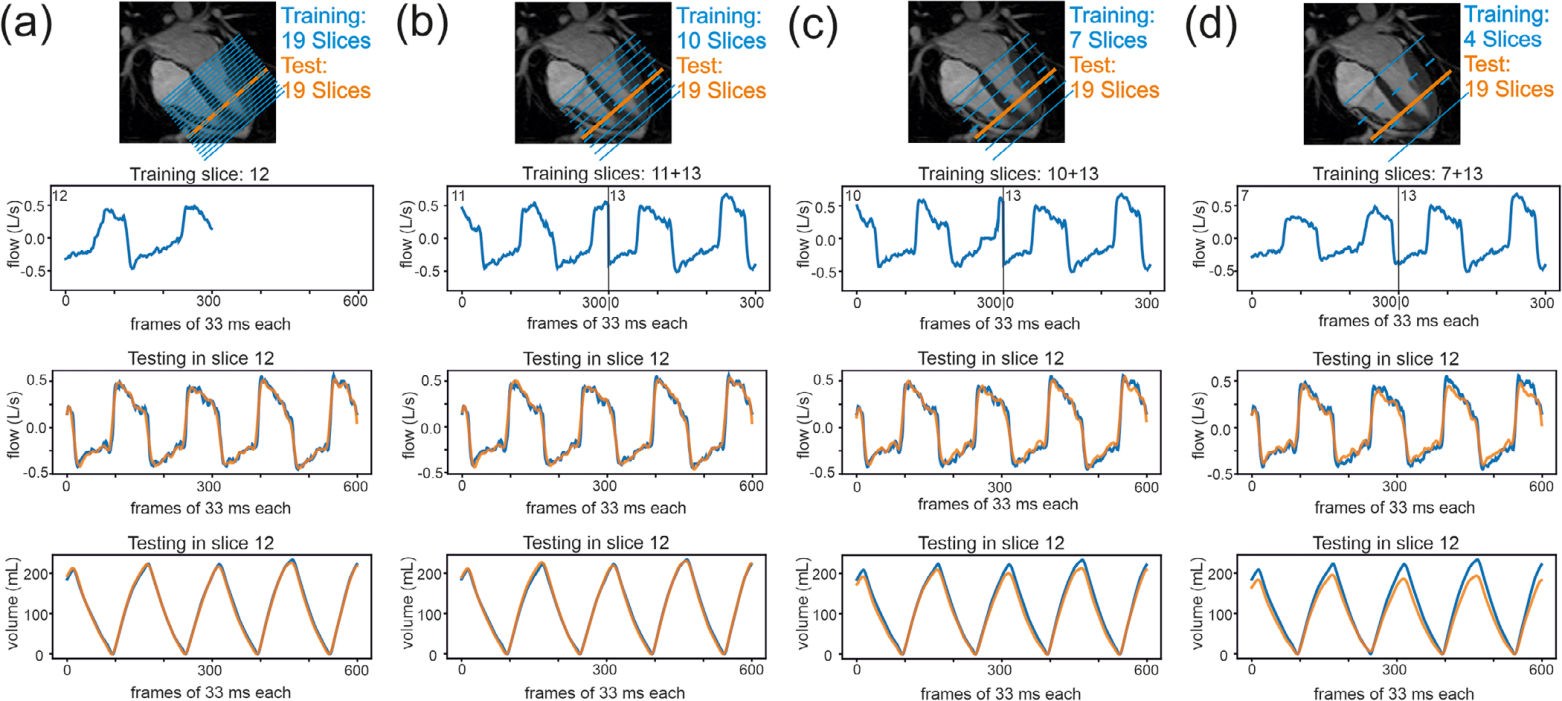
Reducing the number of slices. Comparison of predicted airflow curves (middle row, orange) respectively volume curves (bottom row, orange) with the corresponding measured airflow curves (middle row, blue) and volume curves (bottom row, blue) for a progressively reduced number of slices used for training: (a) training (blue dashed line) and testing in the same slice (orange line), (b) training using the two adjacent slices (blue dashed lines), (c) training using two more distant adjacent slices (blue dashed lines), (d) training using two even more distant adjacent slices (blue dashed lines).

After having reduced the number of required breathing cycles, an additional reduction of the number of slices was tested. Reducing the number of slices to every second slice (i.e., 10 slices) still resulted in an excellent prediction without shift and with good agreement of the peaks (Figure 5a and b). Moreover, no relevant difference of R^2^ variability across predicted slices and subjects was observed (Figure S1 and Figure S2). Reducing the number of slices to every third slice (i.e., seven slices) or every sixth slice (i.e., four slices) resulted in an underestimation of maximal airflow and lung volume (Figure 5c and d). Now, a difference in R^2^ variability between predicted slices and subjects was observed (Figure S1 and Figure S2). Especially, a training with every sixth slice revealed a strong R^2^ variability for adjacent slices (Figure S2d).

Statistical data analysis of cumulative quantitative results of all subjects for testing on complete cardiac volumetries with different training sets confirmed these observations (Table S2 and Figure S3).The combination of a training set reduced to two normal breaths and training in every second (i.e., 10 slices) with a total duration of the scan to 100 s allowed an excellent prediction of volume with a bias of 4.27 mL and a 95% confidence interval (limit of agreement) of ‐24 to 33 mL (Figure S3f) and flow with a bias of ‐0.01 L/s with a 95% confidence interval of ‐0.1 L/s to 0.084 L/s (Figure S3b).

### 3.3 Regional contribution to the model

**FIGURE 6:**
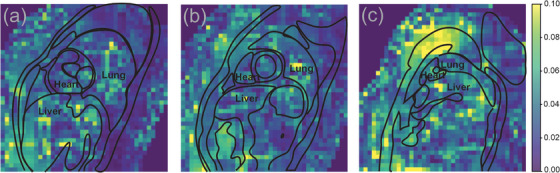
Contributing regions. Weights representing the contribution (0, no contribution, 0.10 high contribution) of the optical airflow of the trained model for one subject of a basal slice (a), a midventricular slice (b), and an apical slice (c).

The distribution of the weights of the trained model for one basal, midventricular, and apical slice of one example subject is shown in Figure 6. The highest weights of the basal slices were localized close to the diaphragm in the caudal part of the thoracic wall (Figure 6a). In the apical slices the areas of heavier weights shifted towards the apical part of thoracic wall (Figure 6c). The prominent weights in midventricular slices showed a more homogeneous distribution around the thoracic wall and diaphragm as illustrated in Figure 6b.

### 3.4 Training set reusability on unknown subject

**FIGURE 7:**
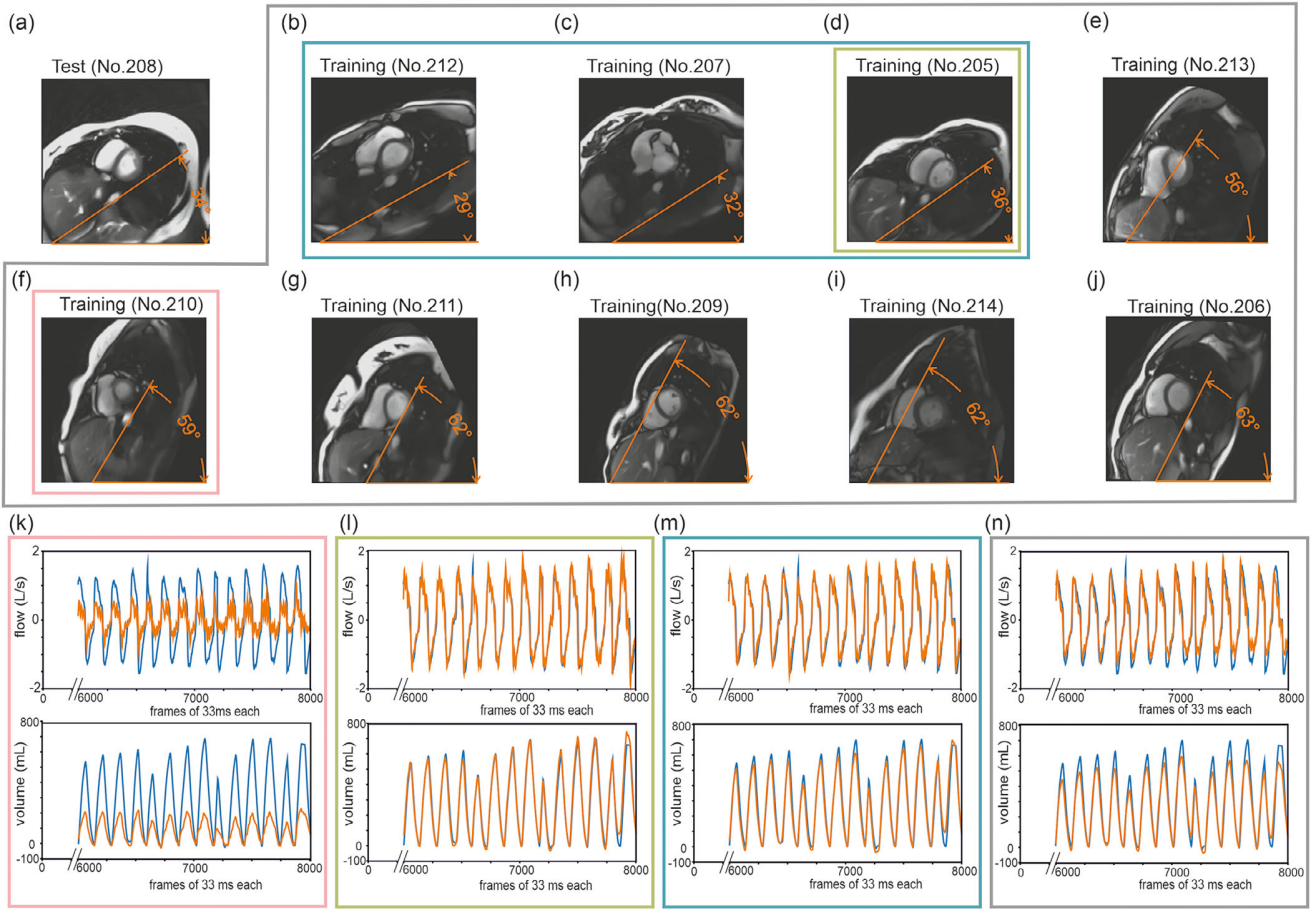
Unknown subject. Measured data (blue lines) and predicted values (orange lines) for airflow (second last row) and volume (last row). Image orientation by degree of thoracic tilt (angle in orange) is displayed for each recorded subject (a‐j). Prediction in the tested subject is poor with different image orientation (k, pink outline), is excellent if the image orientation was similar to the trained subject (l, green outline), is similarly excellent by training with multiple subjects with similar image orientation (m, turquoise outline). Increasing the number of subjects used for training without selecting for image orientation did not improve the prediction in the tested subject (n, gray outline).

Prediction based on a model that was trained with different subjects yielded good results when the image orientation was similar and could even be increased by rotating testing images to align with the image orientation of the trained subject (Figure S4) or by training on a higher number of subjects when image orientation was similar (Figure 7 and Table S3). The influence of height and weight on the quality of prediction was not important (data not shown).

### 3.5 Training set reusability on a second scan of the same subject

**FIGURE 8:**
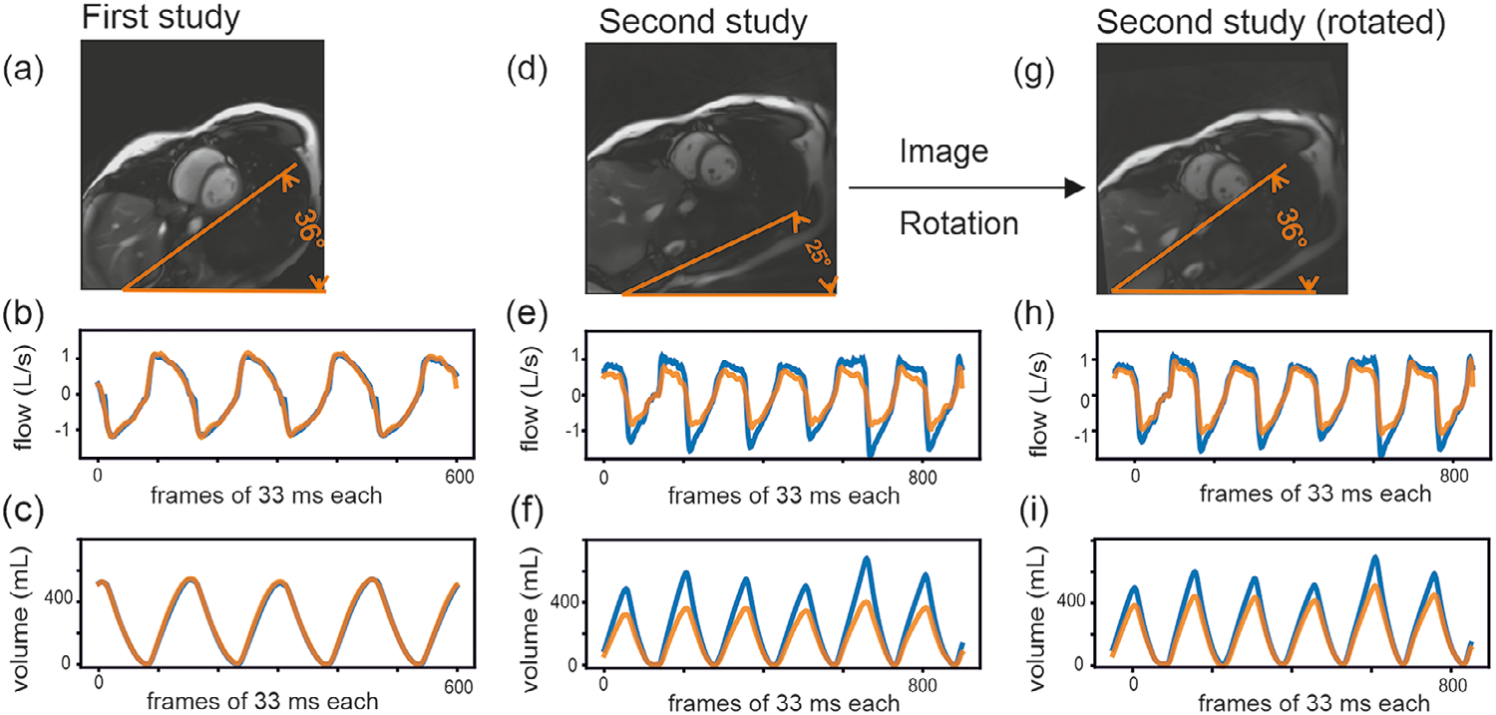
Test‐retest reliability. Comparison of the performance of the algorithm obtained after training with the optimized protocol, two breaths per slice and every second slice, in a subject that was recorded twice (first study: (a‐c), second study (d‐f)). A secondary comparison was performed on the later recording which was rotated to align with the thoracic tilt (angle in orange) of the first study (second study rotated (g‐i)).

The reuse of the training set of the first scan on a second scan of the same subject recorded at a different time allowed for a differentiation between inspiration and expiration but suffered to predict peaks of airflow and lung volume properly (Figure 8). However, the orientation of the slices was different in the two studies (Figure 8a and d).

Similarly, the quantitative analysis revealed a reasonable correlation but a poor prediction regarding quality criteria (Table S5). Since scaling was the main problem, the difference between predicted and measured aTV got poorer with increasing mean of predicted and measured aTV (Figure S5e). Rotating the second study according to the image orientation of the first study (Figure 8g), improved the qualitative and quantitative prediction (Figure 8, Table S5 and Figure S5).

## 4 DISCUSSION

Spirometry is the gold standard to assess lung volume and airflow quantitatively. Both parameters are required to study heart‐lung interactions under normal physiological conditions as well as in patients.

Our previous studies^4^, ^12^ already demonstrated the feasibility of performing spirometry within the active MR scanner. Not only could we show that the quantitative respiratory information provided by spirometry can be used for respiratory binning and image stabilization, but we also found that it enables the assessment of heart‐lung interactions and even the Frank‐Starling mechanism.^4^

Previous studies suggest that the invasively measured pleural pressure is probably the best parameter to quantify the effect of respiration on the cardiovascular system.^13^

Esophageal pressure monitoring with a nasogastric tube could provide a surrogate marker for the intrapleural pressure.^14^

However, esophageal pressure measurements suffer from their invasiveness and their significant technical and procedural challenges.^15^ Since the intrapleural pressure is closely related to the lung volume during spontaneous breathing,^16^ currently, we consider spirometry to be the best non‐invasive parameter to estimate the impact of breathing on the cardiocirculatory system.

MRcS is relatively easy to perform and generally well tolerated. However, in our experience, the use of a tight‐fitting mask can become uncomfortable during prolonged studies. This is particularly true for young children, individuals with severe obesity, those with preexisting respiratory conditions such as asthma, and patients with cardiac conditions like heart failure. Additionally, people who experience psychological distress—such as claustrophobia or anxiety disorders—may find wearing the mask especially challenging.

Allowing the mask to be removed while still extracting important respiratory information from the MR images could enhance patient comfort. It can be expected that removing the mask would improve compliance and perhaps even enhance the accuracy of the measurements.

In this study, we demonstrated that—after a short learning phase—these quantitative data can be provided by the analysis of the MR images, without continuing spirometry.

For this purpose, a ridge regression model was trained on the optical flow estimated from MR images.

In this paper we demonstrated that reliable quantitative spirometry data can be provided by a learning phase as short as two normal breaths per slice and using a reduced number of ten slices  instead of training all the slices. The procedure resulted in a scan time of 100 s for learning sufficiently to provide highly accurate quantitative data for subsequent complete volumetries. Including deep breaths in the learning phase did not improve but even deteriorated the prediction, probably because the additional information was not relevant for the subsequent normal breathing pattern.

A further reduction in the number of slices resulted in qualitatively good but semiquantitative data, as was also the case when using a trained model in other studies of the same or unknown subjects. Improving the reproducibility of the orientation of the short‐axis stack is probably the most critical factor for enhancing accuracy, and this cannot be substituted merely by increasing the number of studies included in the model.

We could show that the information was largely provided by the movement of the thoraxes and diaphragms and not by the pulmonary signals. Without prior spirometry, similar methodological challenges can be expected as with other indirect methods for quantifying respiration.

The possible applications are diverse. If qualitative data is sufficient, the method described is probably an alternative to existing options to monitor breathing,^17^ for example, respiratory bellows, belts, navigator echoes^18^ or self‐gating techniques.^19^ A pre‐trained model could be used in the same way as previously used methods and classify the breathing phases completely without additional equipment and without spirometry to allow motion control and even an orienting comparison of respiratory‐dependent changes. Since no additional MR measurements, for example, during navigator echoes, are required, continuous imaging is still possible.

However, the decisive advantage of the method is not only the control of movement, but rather the provision of quantitative data on lung function.

Examples of scientific questions are studies on physiology in which heart‐lung interactions are examined, most of which have been performed using qualitative techniques.^20^, ^21^ Clinically relevant are also diseases in which breathing plays a major role in the pathophysiology of the disease (e.g., chronic thromboembolic pulmonary hypertension,^22^ right ventricular dysfunction,^12^ heart failure with preserved ejection fraction (HFpEF)^23^).

The method should also be particularly helpful in examinations in which the short‐axis stack is carried out several times in the same examination, that is, stress examinations, for example, ergometry or in pharmacologically induced stress tests.

The main limitation of the proposed method was that, despite the possibility of significantly shortening the time with spirometry and thus improving patient comfort, a short period of spirometry remained necessary for quantitative analysis. In addition, while the application of a trained model to a subsequently acquired test set, either from the same subject or from an unknown one, provided qualitative information, it did not yield quantitatively reliable results. Even if the effort required for spirometry is not different from spirometry outside of the MR, it is not as easy to implement in the context of a radiological examination. In addition, the training and the application of this method to the rest of the examination require additional work and calculation steps in post‐processing, so that the result is not available during the examination but only afterwards. Furthermore, it will be necessary to demonstrate that this technique can also be used at 3T and with other fast imaging sequences. It is still unclear whether the performance would be similar with other protocols, for example, different plane orientations or in other age groups.

## 5 CONCLUSION

In conclusion, a shortened period with MRcS can provide quantitative data for the study of heart‐lung interactions. This will be especially useful in subjects who have problems with MRcS and when several volumetric studies are part of the protocol, such as stress MRI studies. If semi‐quantitative data are sufficient, for example, to define expiration versus inspiration, for image stabilization or to characterize the respiratory phase for binning, this technique can be considered equivalent to other techniques monitoring respiration (e.g., navigator echoes, respiratory belts).

## CONFLICT OF INTEREST STATEMENT

J. Frahm and D. Voit are co‐inventors of a patent and software describing the real‐time MRI technique used here. The other authors have no relevant conflicts of interest to disclose.

## Supporting information



Supporting information

Supporting information
