# Supplementary material for: Machine‐learning‐based prediction of respiratory flow and lung volume from real‐time cardiac MRI using MR‐compatible spirometry
Source: Med Phys. 2025 Aug 11;52(8):e18019. doi: 10.1002/mp.18019 (PMC12340473; doi:10.1002/mp.18019)
Supplement: Supplementary file 1 — Supporting information [file MP-52-0-s001.docx]

**Table S1**

| **Training Set** | **Volume** | | | | **Airflow** | | | |
| --- | --- | --- | --- | --- | --- | --- | --- | --- |
| **Type/ Number of Breaths** | **R^2^**  mean±SD [min-max] | **rMSE**  mean±SD [min-max] | **aTVD [mL]**  mean±SD [min-max] | **QC_vol_** | **R^2^**  mean±SD [min-max] | **rMSE**  mean±SD [min-max] | **aMFD [mL/s]**  mean±SD [min-max] | **QC_flow_** |
| 4 deep + 4 normal | **0.993**±0.003  [0.989-0997] | **0.003**±0.002 [0.001-0.007] | **7**±2  [3-11] | 10/10 | **0.986**±0.005  [0.977-0.994] | **0.017**±0.009 [0.006-0.026] | **34**±20 [13-85] | 10/10 |
| 4 deep | **0.961**±0.026 [0.916-0.996] | **0.009**±0.008 [0.001-0.030] | **16**±10 [5-36] | 6/10 | **0.961**±0.034  [0.871-0.994] | **0.034**±0.028 [0.007.0.109] | **47**±20 [16-76] | 10/10 |
| 4 normal | **0.993**±0.003 [0.987-0.997] | **0.002**±0.001 [0.001-0.004] | **7**±2 [4-11] | 10/10 | **0.986**±0.006 [0.974-0.994] | **0.013**±0.004 [0.005-0.019] | **24**±8 [14-37] | 10/10 |
| 2 normal | **0.981**±0.018 [0.937-0.996] | **0.005**±0.004 [0.001-0.009] | **11**±6  [6-25] | 9/10 | **0.978**±0.012  [0.954-0.989] | **0.019**±0.007 [0.010-0.035] | **35**±18 [19-86] | 10/10 |
| 1 normal | **0.975**±0.026 [0.969-0.996] | **0.012**±0.013 [0.002-0.037] | **17**±9 [8-36] | 7/10 | **0.959**±0.035 [0.859-0.986] | **0.032**±0.014 [0.014-0.057] | **40**±13 [23-65] | 10/10 |
| 0.5 normal | **-0.762**±1.372 [-2.585-0.919] | **0.496**±0.393 [0.027-1.098] | **84**±52 [23-167] | 0/10 | **-0.383**±1.124  [-2.084-0.881] | **0.441**±0.239 [0.091-0.821] | **76**±39 [26-147] | 8/10 |

**Descriptive statistical data on predictive information of midventricular slices. Models trained with different breathing patterns and number of breaths.**

µ, mean, *SD*, standard deviation and minimum-to-maximum range of *R^2^*, coefficient of determination, *rMSE*, relative mean squared error, *aTVD*, absolute tidal volume difference in mL and *aMFD,* of the absolute maximal airflow difference in mL/s of all subjects (N=10, 1 slice per subject with 18 breaths per slice). QC_volume_, number of subjects fulfilling clinical quality criterion for volume (*aTVD_s_*, mean aTVD for one subject, <20mL) and QC_flow_, number of subjects fulfilling clinical quality criterion for airflow (*aMFD_s_***,** mean aMFD for one subject, <100 mL/s).

**Table S2**

| **Training Set** | **Volume** | | | **Airflow** | | |
| --- | --- | --- | --- | --- | --- | --- |
| **Number of Slices** | **R^2^**  **µ**±SD [min-max] | **rMSE**  **µ**±SD [min-max] | **QC_volume_** | **R^2^**  **µ**±SD [min-max] | **rMSE**  **µ**±SD [min-max] | **QC_flow_** |
| 19 | **0.991**±0.010  [0.985-0.997] | **0.003**±0.001  [0.001-0.005] | 9/10 | **0.984**±0.007 [0.977-0.989] | **0.015**±0.004  [0.009-0.022] | 10/10 |
| 10 | **0.990**±0.009  [0.983-0.995] | **0.003**±0.001  [0.002-0.005] | 10/10 | **0.984**±0.006 [0.977-0.990] | **0.015**±0.004  [0.009-0.023] | 10/10 |
| 7 | **0.987**±0.013 [0.974-0.995] | **0.004**±0.002  [0.002-0.007] | 9/10 | **0.982**±0.009  [0.973-0.989] | **0.018**±0.005  [0.010-0.026] | 10/10 |
| 4 | **0.967**±0.039 [0.913-0.987] | **0.011**±0.006 [0.004-0.026] | 4/10 | **0.967**±0.023 [0.933-0.982] | **0.029**±0.008 [0.017-0.045] | 10/10 |

**Descriptive statistical data on predictive information of complete cardiac volumetries using different numbers of slices for training.**

*µ,* mean, *SD*, standard deviation and minimum-to-maximum range of *R^2^*, coefficient of determination and *rMSE*, relative mean squared error, for all subjects (N=10, 19 slices per subject with 4 breaths per slice). QC_volume_, number of subjects fulfilling clinical quality criterion for volume (*aTVD_s_*, mean absolute tidal volume difference for one subject, <20mL) and QC_flow_, number of subjects fulfilling clinical quality criterion for airflow (*aMFD_s_***,** mean absolute maximal airflow difference for one subject, <100 mL/s).

**Table S3**

| **Training Set** | **Volume** | | | **Airflow** | | |
| --- | --- | --- | --- | --- | --- | --- |
| **Subject Number** | **R^2^**  ±SD_B_ | **rMSE**  ±SD_B_ | **aTVD**  **[mL]**  **µ**± SD | **R^2^**  ±SD_B_ | **rMSE**  ±SD_B_ | **aMFD**  **[mL/s]**  **µ**±SD |
| 210 (different angle) | **0.55**±0.003 | **0.370**±0.184 | **311**±148 | **-0.08**±0.01 | **0.450**±0.222 | **518**±338 |
| 210 (similar angle after rotation of test images (208)) | **0.89**±0.002 | **0.038**±0.019 | **133**±92 | **0.89**±0.002 | **0.112**±0.055 | **247**±257 |
| 205 (similar angle) | **0.92**±0.002 | **0.027**±0.023 | **55**±59 | **0.92**±0.002 | **0.077**±0.038 | **253**±211 |
| 205, 207, 212 (similar angles) | **0.91**±0.002 | **0.030**±0.015 | **65**±62 | **0.92**±0.001 | **0.079**±0.039 | **228**±218 |
| 205-207, 209-214 | **0.92**±0.002 | **0.038**±0.019 | **126±**91 | **0.90**±0.002 | **0.100**±0.052 | **217**±232 |

**Descriptive statistical data on predictive information of one subject 208 trained with unknown subjects.**

*R^2^*, coefficient of determination, *rMSE,* relative mean squared error and bootstrap *SD_B_*, standard deviation of rMSE and R². *µ,* mean and *SD* of *aTVD,* absolute tidal volume difference in mL and *aMFD,* absolute maximal airflow difference in mL/s for all breaths within cardiac volumetry (19 slices with 6 breaths per slice) of subject 208.

**Table S4**

| **Training Set** | **Volume** | | | | | | **Airflow** | | | | | |
| --- | --- | --- | --- | --- | --- | --- | --- | --- | --- | --- | --- | --- |
|  | **Denormalization with mean and SD of  training subject** | | | **Denormalization with mean and SD of  test subject** | | | **Denormalization with mean and SD of  training subject** | | | **Denormalization with mean and SD of  test subject** | | |
| **Subject Number (No.)** | **R^2^** | **rMSE** | **aTVD**  **[mL]**  **mean** | **R^2^** | **rMSE** | **aTVD**  **[mL]**  **mean** | **R^2^** | **rMSE** | **aMFD**  **[mL/s]**  **mean** | **R^2^** | **rMSE** | **aMFD**  **[mL/s]**  **mean** |
| 205 (similar angle) | 0.92 | 0.027 | 55 | 0.90 | 0.034 | 64 | 0.92 | 0.077 | 253 | 0.92 | 0.081 | 291 |
| 206 (different angle) | 0.92 | 0.028 | 67 | -6.81 | 2.700 | 601 | 0.92 | 0.083 | 272 | -0.58 | 1.580 | 1747 |
| 207 (similar angle) | 0.60 | 0.138 | 173 | 0.85 | 0.052 | 99 | 0.92 | 0.172 | 325 | 0.90 | 0.099 | 212 |
| 209 (different angle) | 0.19 | 0.280 | 270 | 0.13 | 0.300 | 236 | 0.66 | 0.335 | 462 | 0.55 | 0.450 | 917 |
| 210 (different angle) | -0.08 | 0.370 | 311 | 0.56 | 0.153 | 172 | 0.55 | 0.450 | 518 | 0.72 | 0.284 | 228 |
| 211 (different angle) | 0.66 | 0.117 | 181 | -1.50 | 0.864 | 420 | 0.82 | 0.180 | 278 | 0.18 | 0.824 | 1249 |
| 212 (similar angle) | 0.91 | 0.031 | 78 | -0.72 | 0.594 | 362 | 0.92 | 0.078 | 219 | 0.40 | 0.600 | 961 |
| 213 (different angle) | 0.44 | 0.195 | 233 | 0.34 | 0.227 | 178 | 0.75 | 0.252 | 398 | 0.79 | 0.210 | 179 |
| 214 (different angle) | 0.00 | 0.344 | 302 | 0.84 | 0.055 | 73 | 0.63 | 0.374 | 590 | 0.86 | 0.143 | 372 |

**Adaptive denormalization.** **Statistical data on predictive information for one test subject (No. 208) trained with different unknown subjects (No. 205/206/207/209/210/211/212/213/214).**
For normalization mean and standard deviation (SD) of the training subject’s flow data were used. Denormalization was either performed using mean and SD of the flow data from the training subject (previous method) or adaptively using mean and SD of the test subject. Adaptive denormalization showed no coherent improvement compared to denormalization of the previously described method.

*R^2^*, coefficient of determination, *rMSE*, relative mean squared error, *aTVD,* mean absolute tidal volume difference in mL and a*MFD,* mean absolute maximal airflow difference in mL/s.

**Table S5**

| **Training Set** | **Volume** | | | **Airflow** | | |
| --- | --- | --- | --- | --- | --- | --- |
| **Recording** | **R^2^ µ**±SD | **rMSE**  **µ**±SD | **aTVD [mL]**  **µ**±SD | **R^2^**  **µ**±SD | **rMSE**  **µ**±SD | **aMFD [mL/s] µ**±SD |
| First study | **0.995**±0.004 | **0.002**±0.001 | **12**±10 | **0.99**±0.002 | **0.011**±0.003 | **65**±54 |
| Second study | **0.76**±0.20 | **0.085**±0.072 | **148**±81 | **0.70**±0.37 | **0.115**±0.068 | **178**±139 |
| Second study (rotated) | **0.89**±0.09 | **0.039**±0.031 | **98**±54 | **0.89**± 0.09 | **0.065**±0.040 | **111**±84 |

**Descriptive statistical data on predictive information with two timely different recordings of one subject 205.**

The second recording was tested twice, without and with rotation to align with the thoracic angle of the first recording.

*µ,* mean and *SD,* standard deviation of *R^2^*, coefficient of determination, *rMSE*, mean relative mean squared error, *aTVD*, absolute tidal volume difference in mL and *aMFD,* absolute maximal airflow difference in mL/s for the cardiac volumetry (19 slices with 4-6 breaths per slice) of subject 205.

**Video S1**


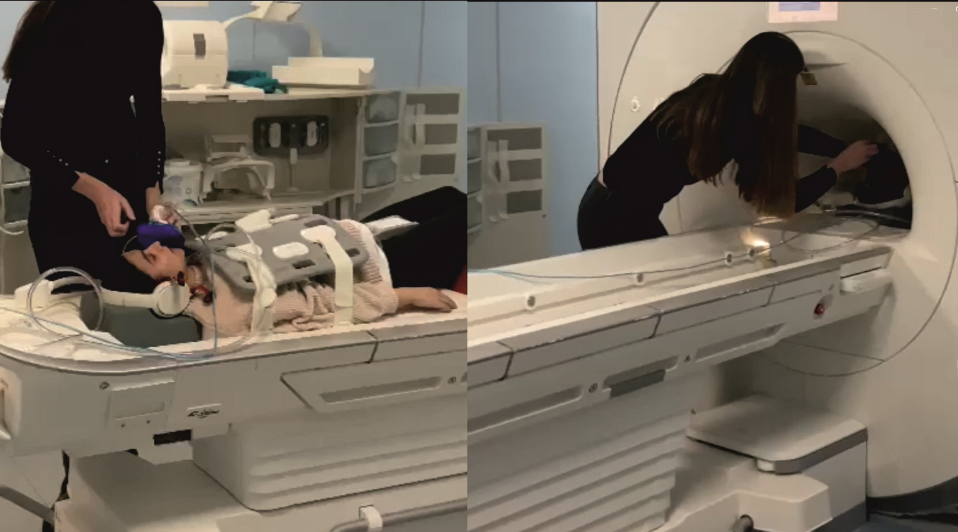


**Removal of the face mask.** Removing the face mask takes only a few seconds and can be performed without moving the MR table.

**Figure S1**


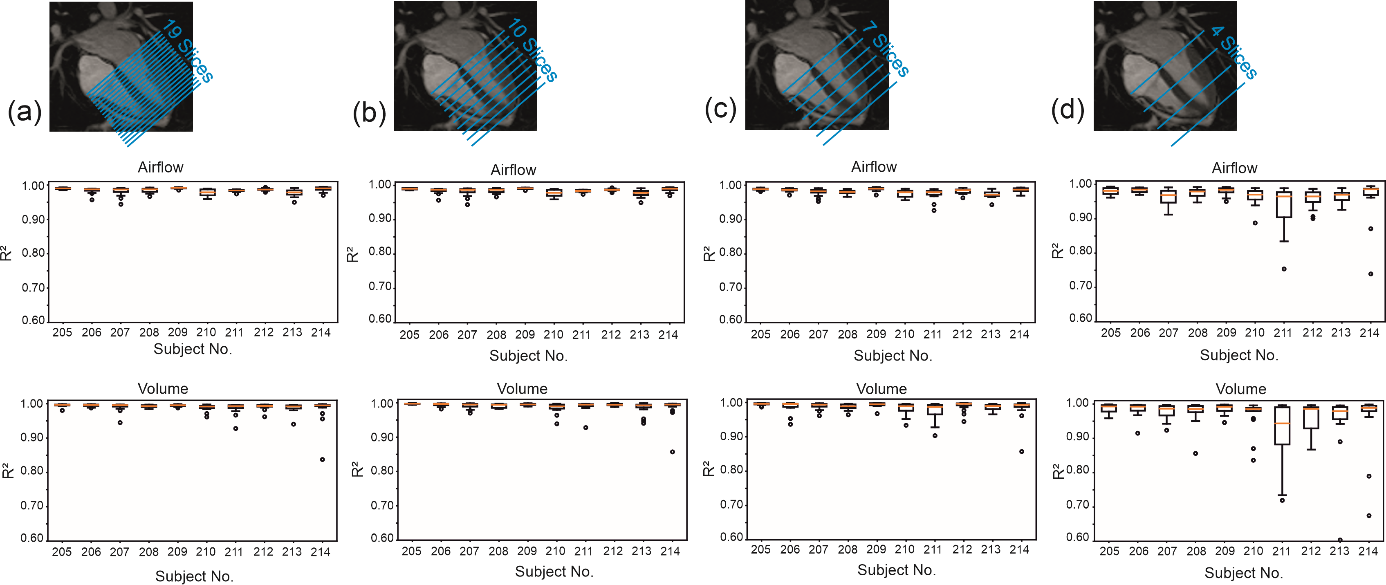

**Variability of coefficient of determination between subjects.** Comparison of coefficient of determination (R²) for predicted airflow (middle row) and predicted volume (bottom row) of each subject for all predicted slices for a progressively reduced number of slices used for training: (a) 19 slices used for training, (b) 10 slices used for training, (c) 7 slices used for training and (d) 4 slices used for training.

**Figure S2**


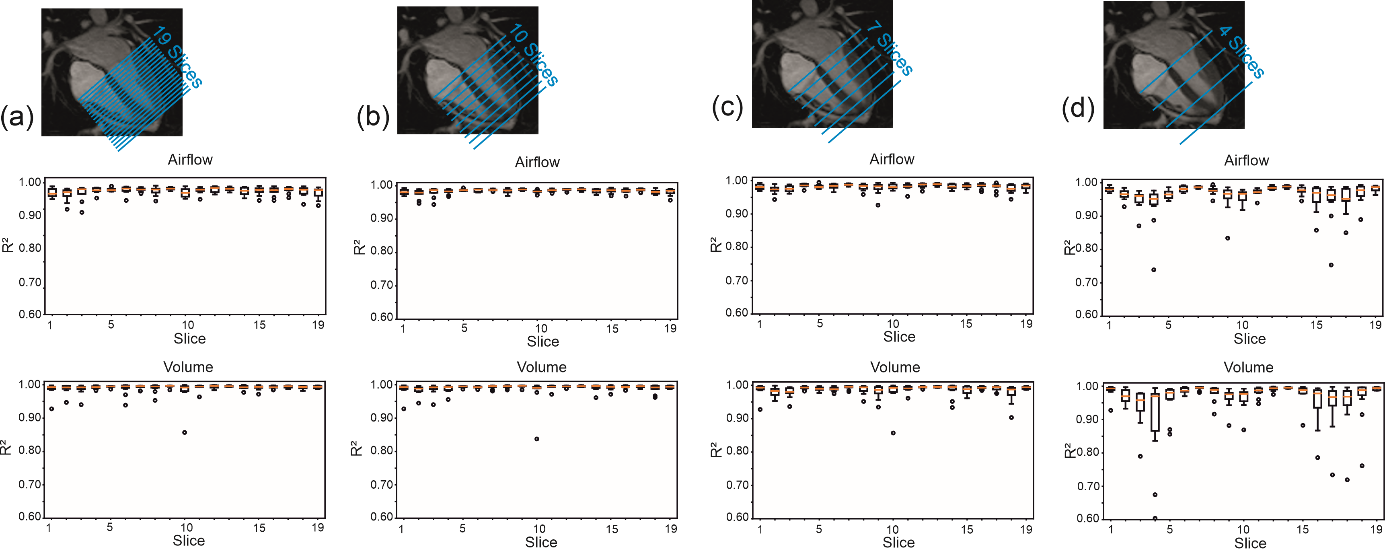


**Variability of coefficient of determination between slices.** Comparison of coefficient of determination (R²) for predicted airflow (middle row) and predicted volume (bottom row) of each predicted slice of all subjects for a progressively reduced number of slices used for training: (a) 19 slices used for training, (b) 10 slices used for training, (c) 7 slices used for training and (d) 4 slices used for training.

**Figure** **S3**


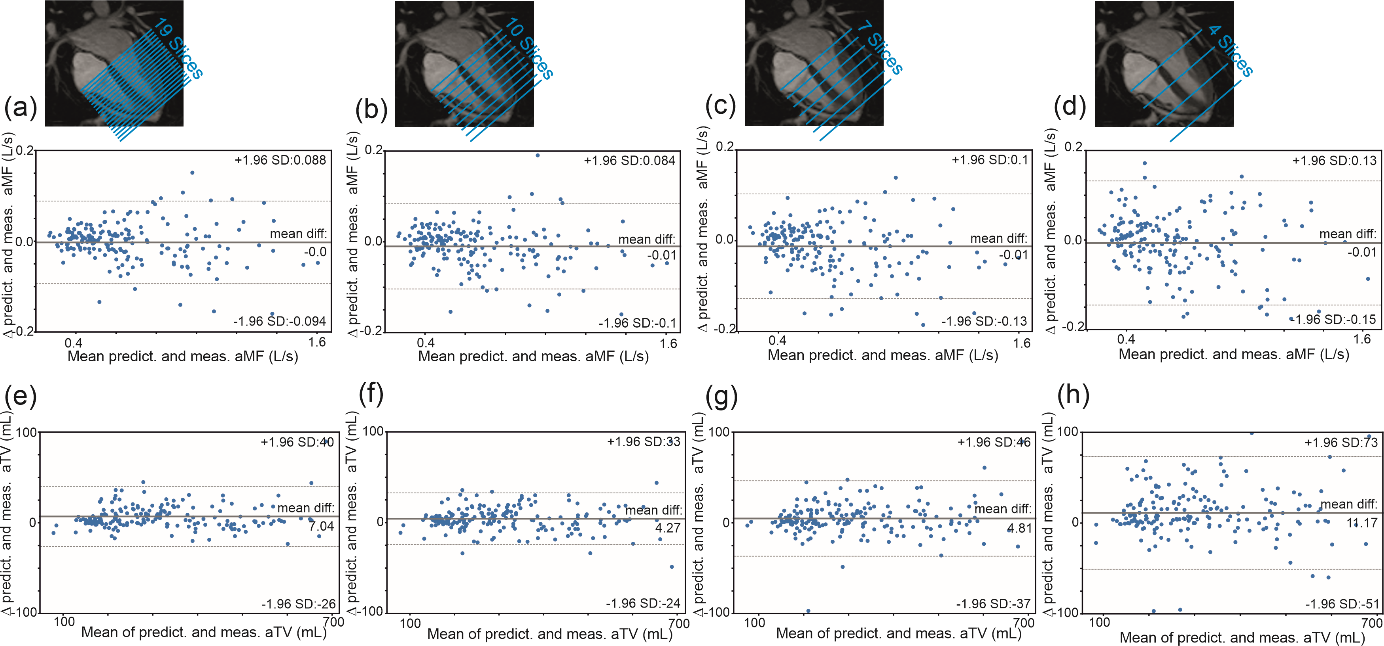


**Bland-Altman (BA) plots for quantitative evaluation of predictive information of complete cardiac volumetries using different numbers of slices for training.** Every BA graph illustrates for every subject and every slice one randomly selected pair of peaks of predicted (predict.) and measured (meas.) data of absolute maximal airflow (aMF) and absolute tidal volume (aTV). Every column represents a different number of slices used for training. The first row of BA graphs displays aMF in L/s for each shortened training set (a-d). Similar bias and limit of agreement (LoA) are seen in training with all slices (a) and training with 10 slices (b), while shortening to seven slices (c) and four slices (d) used for training resulted in broadening of LoA. The second row of BA graphs depicts absolute tidal volume (aTV) in mL for each shortened training set (e-h). Similar bias and LoA are given in (e)-(g). Less than four training slices resulted in broadening of LoA and higher Bias (h).

**Figure S4**

**
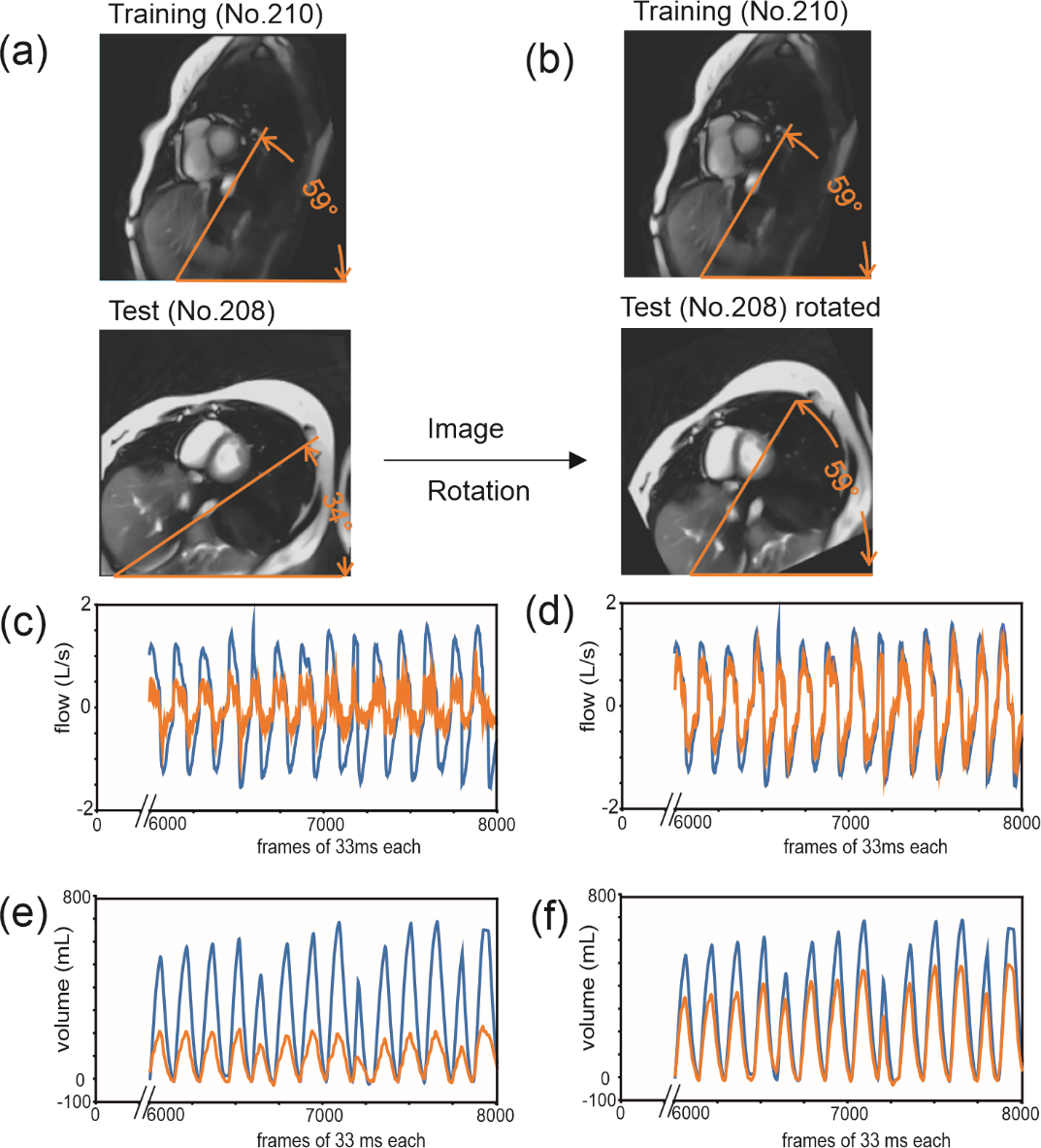
**

**Unknown subject rotated.** Image orientation by degree of thoracic tilt (angle in orange) for the original test set (a) and the rotated test set (b). The latter is rotated to fit to the thoracic tilt of the training set (first row). Measured data (blue lines) and predicted values (orange lines) for airflow (second last row (c,d)) and volume (last row (e,f)) are illustrated.

**Figure S5**

**
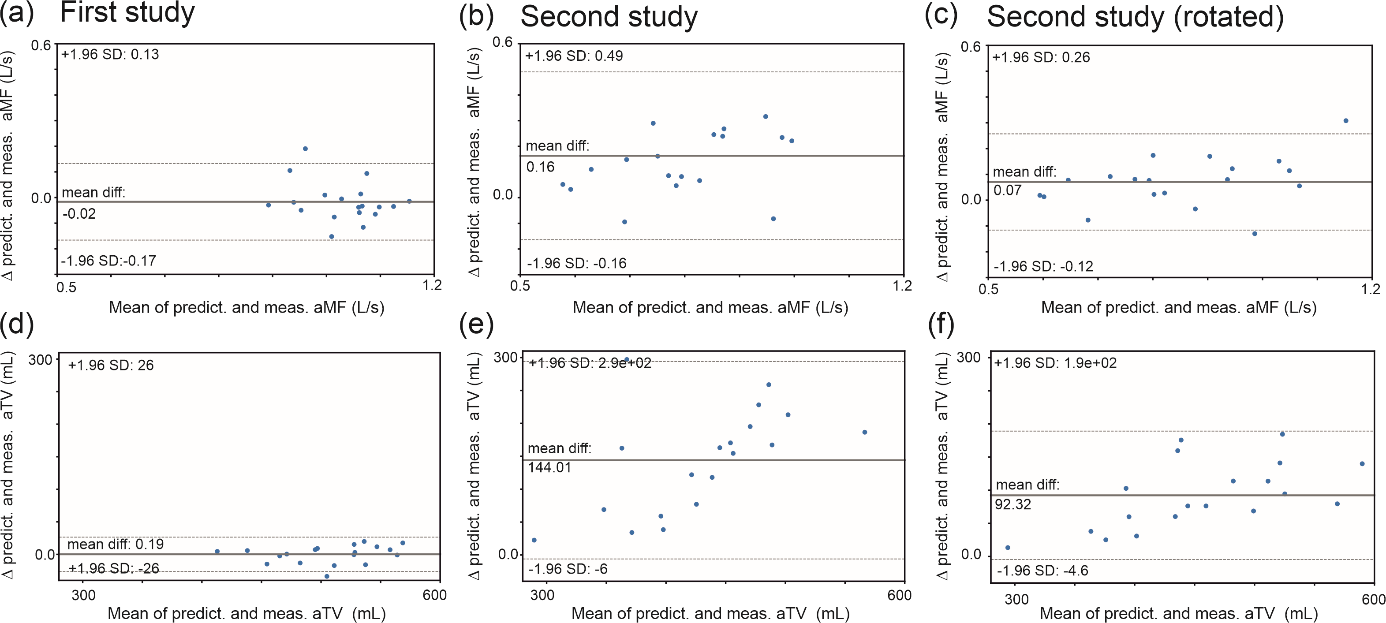
**

**Bland-Altman (BA) plots for quantitative evaluation of predictive information for testing (first study) and retesting (second study) a previously trained model (first study) of one subject.**

For every slice one randomly selected pair of peaks of predicted (predict.) and measured (meas.) data of absolute maximal airflow (aMF) in L/s (a, b, c) and absolute tidal volume (aTV) in mL (d, e, f) is illustrated. Retesting leads to higher bias with higher LoA in aMF (b) and aTV (e). Further, with higher mean of predicted and measured aTV a higher Δ of predicted and measured aTV is seen. By rotating the images of the second study to align with the thoracic tilt of the training set (first study) the bias and LoA of aMF and aTV decrease (c, f).
